# Utilisation of Anthropogenic Food by Red Foxes ( *Vulpes vulpes* ) in Britain as Determined by Stable Isotope Analysis

**DOI:** 10.1002/ece3.70844

**Published:** 2025-02-28

**Authors:** Jonathan W. J. Fletcher, Simon Tollington, Ruth Cox, Bryony A. Tolhurst, Jason Newton, Rona A. R. McGill, Paul Cropper, Naomi Berry, Krishnaveni Illa, Dawn M. Scott

**Affiliations:** ^1^ School of Animal, Rural and Environmental Sciences Nottingham Trent University Southwell UK; ^2^ National Wildlife Management Centre, Animal and Plant Health Agency Gloucestershire UK; ^3^ School of Applied Sciences University of Brighton Brighton UK; ^4^ National Environmental Isotope Facility Scottish Universities Environmental Research Centre, University of Glasgow Glasgow UK; ^5^ Animal and Plant Health Agency York UK

**Keywords:** anthropogenic, diet, red fox, stable isotope analysis, urbanisation, *Vulpes vulpes*

## Abstract

Dietary analyses utilising visual methods to identify stomach and faecal contents have shown that urban red foxes ( *Vulpes vulpes* ) in Britain consume human‐derived (anthropogenic) food to varying degrees. Anthropogenic foods have been implicated in poor health outcomes for synanthropic species that consume them; therefore, it is important to examine the degree of such foods in the British fox diet. We analysed the carbon (δ^13^C) and nitrogen (δ^15^N) stable isotope ratios of whiskers collected from 93 foxes from across Britain to determine: (1) if stable isotope analysis (SIA) distinguished a difference in δ^13^C and δ^15^N between rural and urban foxes, and whether any difference was suggestive of anthropogenic food use; (2) the proportion of anthropogenic food consumption in urban foxes compared to rural foxes using a Bayesian mixing model; (3) whether sex, age or season of collection influenced fox diet as assessed by SIA, in relation to anthropogenic food use. We found the following: (1) urban fox diet was significantly different to rural foxes; urban foxes demonstrated significantly higher δ^13^C and lower δ^15^N, a pattern consistent with anthropogenic food consumption. (2) Food provided either directly or indirectly by humans contributed an estimated 34.6% of urban fox diet compared to approximately 6% of rural fox diet. (3) Across rural and urban foxes combined, there were significant isotopic differences between males and females, with females demonstrating higher δ^13^C. (4) No differences in δ^13^C and δ^15^N between subadults and adults were observed. (5) Season did not have a significant influence on δ^13^C and δ^15^N, despite winter demonstrating the highest δ^13^C and lowest δ^15^N seasonal means. Potential negative outcomes of anthropogenic food consumption are likely to disproportionately impact females more than males and urban‐dwelling foxes more than rural foxes.

## Graphical abstract

Anthropogenic food consumption has been implicated in poor health outcomes for synanthropes. We used stable isotope analysis on British red fox whiskers to show a distinct diet between rural and urban foxes, where rural and urban fox diets consisted of around 6% and 35% anthropogenic food, respectively. Additionally, females rather than males demonstrated isotopic patterns indicative of anthropogenic food use, suggesting that potential health risks from anthropogenic foods are more likely to impact female and urban‐dwelling foxes.

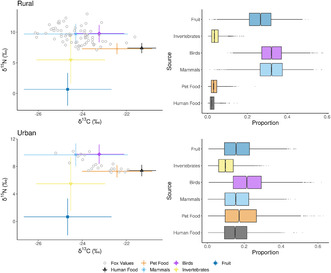

## 1 Introduction

Urbanisation entails the development from natural to human‐inhabited environments (McKinney 2002), associated with high human population densities and infrastructure (e.g., buildings and roads), resulting in broad, impervious surface areas (Wu 2014). In response, some wildlife species avoid these areas whilst other ‘synanthropic’ species can adapt or thrive (Johnston 2001; McKinney 2006). Synanthropes can exploit these environments partly due to the abundance, accessibility and predictability of human‐derived or ‘anthropogenic’ food, including garden and allotment produce, refuse food waste and intentional household provisions such as meal leftovers, pet food and bird food (Bateman and Fleming 2012).

The available food resources for synanthropes may reflect the processed foods found in the industrialised human diet. These processed foods contain high levels of saturated fats, refined grains and sugars, poor micronutrient density and low levels of fibre (Cordain et al. 2005). Ultra‐processed foods are the most extreme form of processing with examples such as bulk‐manufactured bread and reconstituted meats, which have been linked to human obesity (Rauber et al. 2018, 2020). Food resources directly available to synanthropes, such as food waste or commercial foods designed for wildlife consumption, may be nutritionally inadequate (Carpenter and Savage 2021; Gimmel, Eulenberger, and Liesegang 2021). Additionally, some anthropogenic food sources have been implicated in negative health outcomes in a range of wildlife species such as disease occurrence, oxidative stress and associated measures of obesity and insulin resistance (Banks et al. 2003; Schulte‐Hostedde et al. 2018; Leith et al. 2020; Bernat‐Ponce et al. 2023). The use of anthropogenic food presents a nutritional dilemma for synanthropes; however, we must quantify consumption to correctly assess the impact of anthropogenic food on synanthropic species.

The red fox ( *Vulpes vulpes* ), henceforth referred to as ‘fox’, is a mammalian mesopredator with a wide geographic distribution that includes Britain, where they can be found across the urban–rural gradient (Hoffmann and Sillero‐Zubiri 2016; Leckie et al. 1998; Scott et al. 2014). The fox is a generalist omnivore that naturally consumes small mammals, birds, fruit, invertebrates and carrion but can also exploit anthropogenic food (Contesse et al. 2004; Soe et al. 2017). Previous studies estimated that scavenged food (such as food waste and food intentionally left for wildlife) formed 35%–64% of the stomach contents of foxes in London and Bristol (Harris 1981; Saunders et al. 1993) and constituted 37% of the dry weight of fox faecal samples in Oxford (Doncaster, Dickman, and Macdonald 1990).

Scavenged food intake has been shown to vary by season for foxes, with such consumption most prevalent during the winter, a time of low natural food availability (Doncaster, Dickman, and Macdonald 1990; Saunders et al. 1993). Sex differences in anthropogenic food use may also exist; for example, in spring, females spend more time than males feeding in patches of intentional provisioning by households (Dorning and Harris 2017). This likely reflects the seasonal change in foraging behaviour by females to optimise their energy demands during lactation and cub weaning (Fawcett, Fawcett, and Soulsbury 2017; Lanszki et al. 2023). Anthropogenic food consumption may also vary with age due to links with social status. For example, dominant foxes make more within‐territory visits to provisioning households and often utilise available household provisions first compared to subordinates, who may undertake greater extra‐territorial foraging (Dorning and Harris 2017). Additionally, dominant individuals have been shown to live roughly twice as long as subordinates (Baker et al. 1998), putatively skewing anthropogenic food consumption across different age classes. However, the relationship between age and anthropogenic food consumption may not be strictly linear as dominant status may be lost at an older age (Baker et al. 1998). Alternatively, anthropogenic food use across sex and age may differ little due to its overall abundance in various forms, which may lead to indiscriminate consumption between individuals within an urban environment.

To explore anthropogenic food intake in urban foxes, the utility of visual dietary techniques such as stomach or faecal analysis is limited. These ‘visual diet tracing’ methods use hard remains (e.g., hair, bones and feathers) to determine specific food items, but are liable to misidentification and underestimation, at least partly due to the digestibility of food sources (Reynolds and Aebischer 1991; Nielsen et al. 2018). Some forms of anthropogenic food (e.g., food waste such as scavenged meat, bread and other processed foods) are highly digestible and therefore more easily identified in fox stomach contents than in faecal samples (Balestrieri, Remonti, and Prigioni 2011). However, stomach analyses come at the cost of destructive sampling and the risk of uninformative empty stomachs, whereas faecal sampling is less invasive, even though it involves non‐random samples that cannot be attributed to individuals (Darimont and Reimchen 2002). Both methods only provide a temporal snapshot of diet, representing items consumed over a short period of time of a day or so (Nielsen et al. 2018). Studies using these methods may not truly encapsulate the extent of anthropogenic food use in the urban red fox diet.

Stable isotope analysis (SIA) is a diet tracing method that provides a more efficient, potentially non‐destructive and often non‐invasive examination of diet over longer time periods (Newton 2010; Nielsen et al. 2018). The isotopic ratios of food items are reflected in those that consume them; therefore, the analysis of carbon (^13^C/^12^C; δ^13^C) and nitrogen (^15^N/^14^N; δ^15^N) isotope ratios from the tissues of a consumer can reveal information about dietary composition (DeNiro and Epstein 1978, 1981; Kelly 2000). The period of dietary composition reflects the time in which the analysed tissue is synthesised; for example, metabolically inert hair samples can reveal dietary information over a span of weeks (Tieszen et al. 1983; Huelsemann et al. 2009). However, SIA is limited to the identification of a small number of isotopically distinct food sources rather than individual food items (Phillips et al. 2014).

SIA has previously been used to determine the consumption of anthropogenic foods in synanthropic mammals (e.g., coyotes [ *Canis latrans* ] and Eastern chipmunks [ *Tamias striatus* ]), due to high corn or maize content ( *Zea mays* ) (Jahren and Kraft 2008; Murray et al. 2015a; Ouellette and Schulte‐Hostedde 2024). Corn utilises C4 photosynthesis, whereas most natural temperate terrestrial plant species utilise the C3 pathway; C3 plants generally have a δ^13^C range of around −35% to −20%, whereas C4 plants have a range of about −18% to −7% (O'Leary 1988); thus, C4 plant consumption results in relatively higher δ^13^C in animal tissues (Smith and Epstein 1971; Newsome et al. 2010). Values of δ^15^N in animal tissues tend to increase with trophic level in food webs (DeNiro and Epstein 1981; Kelly 2000), since many processes result in the selective retention of ^15^N and loss of ^14^N, particularly by excretion of ^14^N‐rich products (Steele and Daniel 1978). However, urban individuals typically demonstrate lower δ^15^N, reflecting a reduction of natural prey consumption within their diet (Newsome et al. 2010).

Previous studies exploring anthropogenic food consumption in red foxes using SIA have suggested a distinct isotopic difference between urban and rural conspecifics. For example, there was a positive relationship between urban metrics and δ^13^C of foxes in Pennsylvania, USA (Handler, Lonsdorf, and Ardia 2020). Urban fox populations in north‐east Germany had distinct isotopic differences (both δ^13^C and δ^15^N) compared to rural populations, with urban foxes demonstrating higher δ^13^C and lower δ^15^N than rural populations (Scholz et al. 2020).

Accordingly, in this study we aimed to answer the following questions:

(1) Does SIA reveal a significant difference in δ^13^C and δ^15^N between urban and rural foxes in Britain, indicating a distinction in anthropogenic food consumption?

(2) What is the proportional consumption of anthropogenic food among urban and rural British foxes based on SIA?

(3) Is the British fox diet significantly influenced by sex, age and/or season based on SIA? If so, are these indicative of anthropogenic food consumption?

More specifically, we predicted that anthropogenic food would form a larger part of the urban fox diet than rural fox diet and we therefore expected urban foxes in Britain to have a lower mean δ^15^N and a higher mean δ^13^C, as seen in urban foxes from other countries (e.g., Scholz et al. 2020). Furthermore, while fox sex and age may differentially affect anthropogenic food consumption, the overall abundance and accessibility of different anthropogenic foods in urban environments (e.g., food waste) may negate any discernible effects of these factors. Therefore, we predicted that there would be no significant influences of either sex or age on anthropogenic food consumption. As previous fox dietary studies demonstrated higher anthropogenic food consumption during winter (e.g., Saunders et al. 1993), we also predicted that SIA would reveal higher δ^13^C and lower δ^15^N in winter compared to other seasons.

## 2 Material and Methods

### 2.1 Dietary Analysis

Vibrissae samples were collected from a total of 93 fox carcasses sourced by the Animal and Plant Health Agency (APHA) as part of the annual monitoring of the cestode parasite (*Echinococcus multilocularis*) in the British fox population. The ethical review was deemed favourable by the Nottingham Trent University Ethical Review Committee. Georeferenced samples were collected between April 2021 and January 2023 via a network of gamekeepers and landowners. Carcasses were frozen and defrosted at 4°C before examination where sex and age (subadult: < 12 months; adult: ≥ 12 months) were determined, the latter via tooth analysis (Giraudoux, Romig, and Eckert (2001)). No cubs (< 6 months of age) were collected. Date of death was used to assign season to each carcass: spring (March–May), summer (June–August), autumn (September–November) and winter (December–February). Up to five of the longest vibrissae from each fox carcass were plucked and sent to Nottingham Trent University where they were stored at 5°C until further analysis.

Preparation of vibrissae was conducted incorporating recommendations from SUERC (Scottish Universities Environmental Research Centre). Vibrissae samples were initially cleaned using a 2:1 chloroform:methanol solution and air‐dried for 24 h. The base of each vibrissa was cut to lengths of between 3 and 5 mm to achieve individual sample weights of ~0.5 mg. The growth rates of red fox vibrissae have been estimated at 0.32 mm/day (Jacquier et al. 2020); therefore, each sample represented between 9.3 and 15.5 days of previous dietary information. Each sample was placed in a tin capsule (5 × 3.5 mm) and sent for consecutive C and N SIA at SUERC. A continuous‐flow mass spectrometer (Delta Plus XP; Thermo Scientific, Bremen, Germany) and elemental analyser (vario PYRO cube; Elementar, Langenselbold, Germany) were used to ascertain the isotope ratios of C and N from each sample. Three internal standards (GEL, ALAGEL and GLYGEL) were used after every 10 samples to maintain instrumental validity. Isotope ratios were denoted as δ values (δ^13^C/ δ^15^N) in permil (%) based on the equation:

$$\delta^{13}C\;\text{or}\;\delta^{15}N=\left[\left(R_{\text{sample}}/R_{\text{standard}}\right)-1\right]$$

where *R*_sample_ is the respective ratio of ^13^C/^12^C or ^15^N/^14^N and *R*_standard_ is the international references of Vienna PeeDee Belemnite and atmospheric N_2_ for carbon and nitrogen, respectively.

To identify the proportional contribution of different food types in the fox samples, we included six broad food groups from the published fox diet literature encompassing both anthropogenic foods and natural diet items, these were: human food, pet food, mammals, birds, invertebrates and fruit (Harris 1981; Contesse et al. 2004; Soe et al. 2017). These represented only potential food sources, in which the data were not collected directly, but the information regarding the δ^13^C and δ^15^N of each food group was derived from the published literature or by requesting data from the authors (Table 1).

**TABLE 1:** The food resources used in the stable isotope mixing model and their isotopic values.

Food resource	Stable isotope values (SD)	Details	References
Human food	δ^13^C = −23.32% (0.62) δ^15^N = 4.11% (0.81) (*n* = 58)	Values calculated by subtracting human hair TDFs (δ^13^C = 2.50%; δ^15^N = 5.15%) from the mean stable isotope values of British human hair. (These values were: δ^13^C = −20.82% (SD = 0.62), δ^15^N = 9.26% (SD = 0.81).)	O'Connell and Hedges (1999); O'Connell et al. (2012); Bol, Marsh, and Heaton (2007); O'Connell et al. (2001)
Pet food	δ^13^C = −24.42% (1.58) δ^15^N = 4.00% (0.87) (*n* = 167)	Various British brands of wet and dry cat food. No stable isotope data were available for British dog food brands.	Cecchetti et al. (2021)
Mammals	δ^13^C = −26.29% (2.35) δ^15^N = 6.40% (1.64) (*n* = 164)	British mammal species, including: *Myodes glareolus*, *Sorex araneus* , *Microtus agrestis* , *Rattus norvegicus* , *Oryctolagus cuniculus* , *Apodemus sylvaticus*	Cecchetti et al. (2021)
Birds	δ^13^C = −25.23% (1.12) δ^15^N = 6.48% (1.40) (*n* = 61)	British bird species, including: *Turdus merula* , *Sylvia atricapilla* , *Parus caeruleus*, *Pyrrhula pyrrhula* , *Fringilla coelebs* , *Parus ater*, *Prunella modularis* , *Carduelis carduelis* , *Parus major* , *Passer domesticus* , *Columba livia* , *Erithacus rubecula* , *Turdus philomelos* , *Sturnus vulgaris* , *Phylloscopus trochilus* , *Troglodytes troglodytes*	Cecchetti et al. (2021)
Invertebrates	δ^13^C = −26.52% (1.55) δ^15^N = 2.19% (3.89) (*n* = 249)	British invertebrate species, including: *Pterostichus madidus*, *Abax parallelepipedus*, *Nebria brevicollis* , *Arion ater* sp., *Geotrupes stercorarius* , *Noctuidae*, *Tipulidae*, larvae (*Tipulidae* and *Noctuidae*), *Arianta arbustorum*, *Capaea hortensis*, *Capaea nemoralis*, *Lumbricus terrestris*	A. Robertson (Pers. Comm, based on Robertson (2012))
Fruit	δ^13^C = −26.66% (1.98) δ^15^N = −2.63% (2.67) (*n* = 95)	British fruit species, including: *Prunus* sp., *Rubus fruticosus* , *Taxus baccata* , *Pyrus* sp., *Malus* sp., *Rosa* sp.	A. Robertson (Pers. Comm, based on Robertson (2012))

### 2.2 Trophic Discrimination Factors and Stable Isotope Values of Dietary Resources

To account for the physiological enrichment of C and N isotopes in consumers from dietary sources, trophic discrimination factors (TDFs) (i.e., the difference in isotopic values between food source and consumer tissue) must be applied (Phillips et al. 2014). TDFs have been calculated for various red fox tissues (Roth and Hobson 2000), but do not exist for vibrissae. However, TDF values for wolves ( *Canis lupus* ), a related canid species, suggest very similar values between fur and vibrissae (McLaren, Crawshaw, and Patterson 2015). The red fox TDF values for C and N (i.e., Δ^13^C and Δ^15^N) determined by Roth and Hobson (2000) were generated from samples taken from captive fur‐farmed foxes fed on a single pellet diet. However, Δ^13^C values can vary due to factors such as dietary source (i.e., C3 or C4 plant source), which we expect to be mixed in the context of natural and anthropogenic food (Stephens et al. 2022). We therefore used the 2.0% (±0.2 SD) and 3.3% (±0.2 SD) TDF values determined by Stephens, Shipley, and Moll (2023) for Δ^13^C and Δ^15^N, respectively. These values are similar to Roth and Hobson (2000), 2.6% for Δ^13^C and 3.3% for Δ^15^N, although the error terms were not clearly provided.

### 2.3 Measuring Urbanisation

**FIGURE 1:**
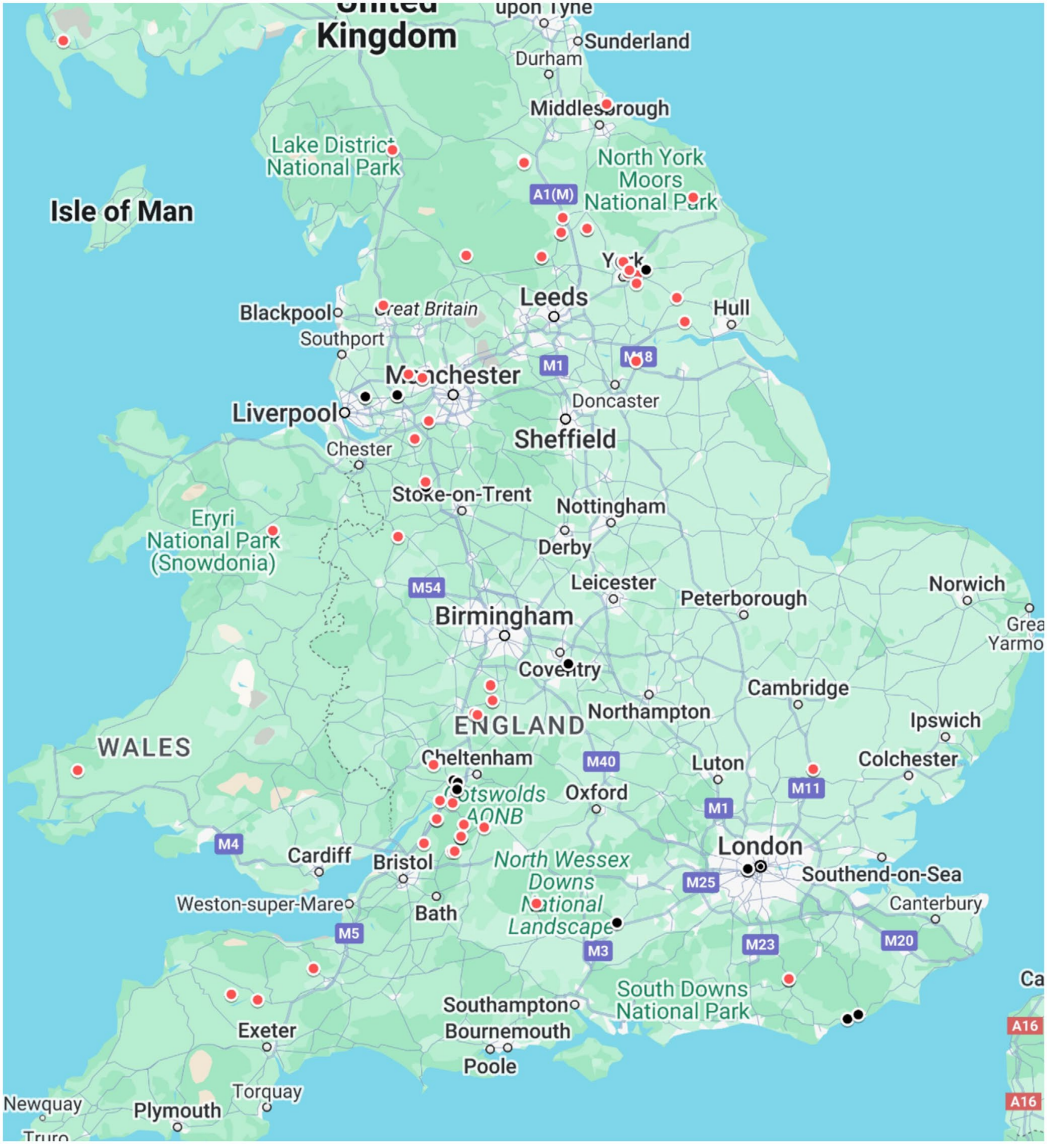
Original rural (red) and urban (black) fox locations. Google Maps. Map of Britain. Retrieved November 11th, 2024, from https://google.com/maps.

Impervious surface density (ISD), which measures the percentage of sealed surface areas such as roads and buildings, is a common proxy measure for urbanisation (Wu 2014). We therefore used ISD to determine urbanisation within a 500 m radius of the location of each fox carcass following Scholz et al. (2020), which also allows for our results to be comparative to this study. Mean ISD was determined per sample using a 2018 COPERNICUS raster map with a resolution of 20 m (https://land.copernicus.eu). As defined by Scholz et al. (2020), individuals were considered ‘urban’ if ISD was ≥ 25% and ‘rural’ if < 25%. This resulted in 23 of 93 samples classified as ‘urban’ (Figure 1).

A six‐figure Ordnance Survey grid reference of each carcass location was provided by the APHA, the centre of the grid location formed the origin from which a circle with a 500 m radius was drawn. The resulting area covered 0.79 km^2^, which acknowledged fox range variability and the tendency for smaller urban ranges. For example, fox range was 0.75 km^2^ at the highest human population densities (Main et al. 2020). Other research has noted even smaller urban home ranges (e.g., 0.14 km^2^; Tolhurst et al. 2016). We assumed foxes were resident rather than dispersing or itinerant, since most dispersing individuals are sub‐adult males during autumn and winter periods (Harris and Trewhella 1988).

Lastly, since some of the foxes originated from similar locations, we grouped datapoints from foxes that were within 500m and killed within seven days of each other to control for a lack of independence between these points. We believe these criteria sufficiently account for any non‐independence both spatially, as it accords with our defined fox home range, and temporally, since a minimum of seven days should reflect an adequate difference in diet. A separate 'Location ID' category was formed in which 34 of 93 datapoints were placed into 13 groups (*n* = 72).

### 2.4 Statistical Analysis

All statistical analyses, other than permutational multivariate analyses of variance (PERMANOVAs), were conducted in R Version 4.3.2 (R Core Team 2023). PERMANOVA tests were run in MATLAB (2023) using the Fathom Toolbox (Jones 2017). To answer our first and third objectives, we used Linear Mixed‐Effect Models (LMMs) on each of δ^13^C and δ^15^N separately, which included the (fixed effect) predictor variables of location (rural and urban), sex (male and female), age (< 12 and ≥ 12 months) and season (spring, summer, autumn and winter). Location ID was included as a random effect to control for the geographical non‐independence of some datapoints.

Following data exploration and model‐checking, the response variable in each case was modelled with a Gaussian distribution and an identity link function. We used the *lme4* and *lmerTest* packages in R to conduct the LMMs (Bates et al. 2015; Kuznetsova et al. 2017). Overall, there were three missing data points, one for age and two for season. To answer the second objective, to determine the extent of anthropogenic food consumption compared to other food resources in urban and rural foxes, we estimated the proportional relative contribution of each food group in their respective diets using *SIMMR*, a Bayesian isotope mixing model R package (Govan et al. 2023). The mixing model included the TDFs (and their standard deviations), the isotope data from our fox samples and the δ^13^C and δ^15^N concentrations of each food source to account for differential elemental concentrations that may exist between food sources of omnivorous species (Phillips and Koch 2002). Since human hair was used as a proxy for human food, we used the elemental concentrations as estimated by Hopkins and Ferguson (2012) for this food group. Since TDFs can influence model outputs (Phillips et al. 2014), the TDFs of Roth and Hobson (2000) were included in an additional mixing model to compare both model outputs. Both SIMMR models ran at the default settings of 10,000 iterations and a burn‐in of 1000. The mixing models were checked for successful convergence, all food sources had a value of 1.

To determine the difference in isotopic variation between the food sources, PERMANOVA tests were conducted.

**FIGURE A1:**
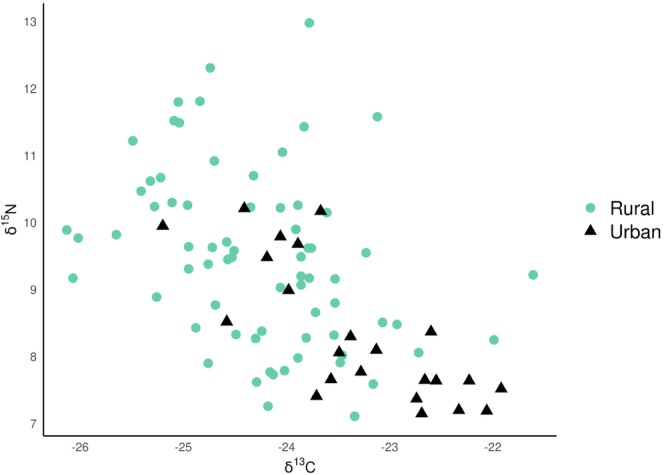
The isotopic data points for rural and urban foxes.

**FIGURE A2:**
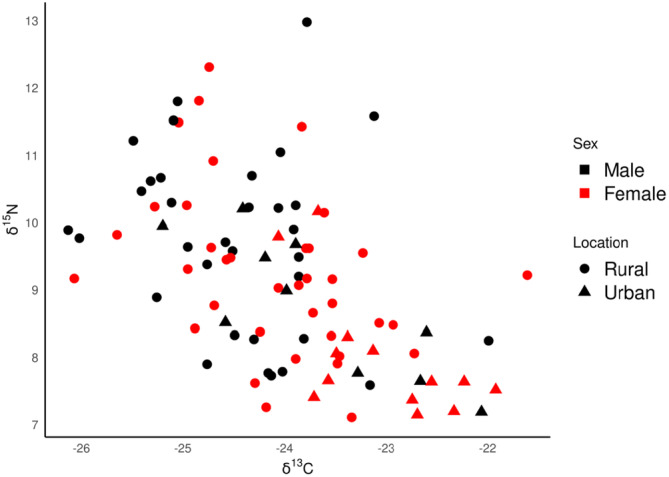
The isotope data points based on both location (shape) and sex (colour).

**FIGURE A3:**
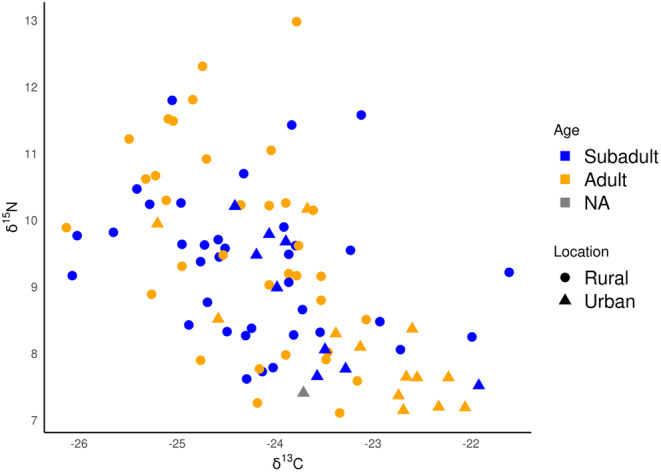
The isotope data points based on location (shape) and age (colour).

**FIGURE A4:**
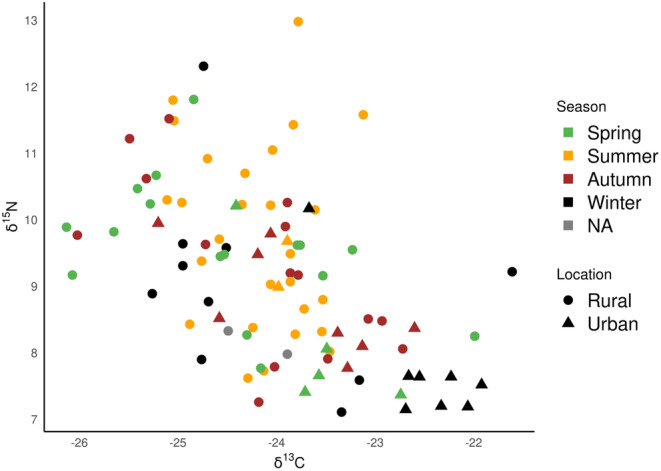
The isotope data points based on location (shape) and season (colour).

Sample sizes and the spread of isotope values for each variable under investigation can be found in the Appendix A. Table A1 and Figures A1, A2, A3, A4.

## 3 Results

Isotopic values of fox vibrissae were: mean δ^13^C = −24.02% (0.96 SD), range = −26.13% to −21.61%; δ^15^N mean = 9.19% (1.33 SD), range = 7.11% to 12.98%.

### 3.1 Location

**FIGURE 2:**
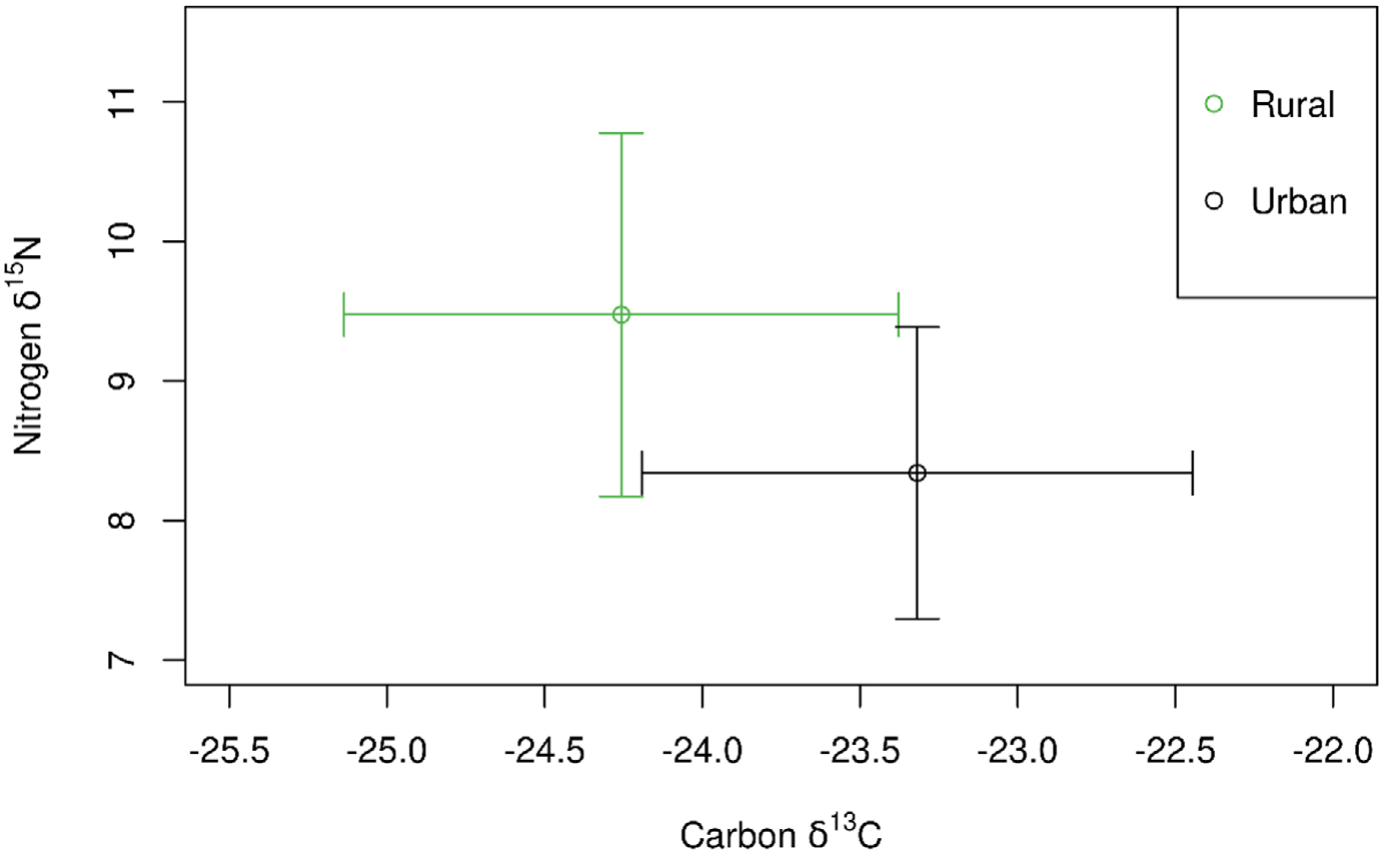
Mean (± SD) δ^13^C and δ^15^N of vibrissae collected from rural (*n* = 70) and urban (*n* = 23) foxes.

For each element, location significantly predicted both δ^13^C and δ^15^N (Table 2), with urban foxes demonstrating higher δ^13^C and lower δ^15^N than rural foxes (Figure 2). The variance for Location ID (random effect) was greater for δ^13^C than δ^15^N (Table 3).

**TABLE 2:** The influence of the fixed effects of location (rural, urban), age, sex and season on each of δ^13^C and δ^15^N from fox vibrissae, using Linear Mixed‐Effect Models (LMMs).

	*β*	SE	*t* Value	*p*
δ^13^C				
Intercept	−25.03	0.43	−57.77	< 0.001
Location	0.89	0.26	3.45	< 0.01
Sex	0.40	0.17	2.37	< 0.05
Age	−0.11	0.18	−0.60	> 0.05
Season				
Summer	0.37	0.27	1.37	> 0.05
Autumn	0.31	0.28	1.09	> 0.05
Winter	0.50	0.32	1.56	> 0.05
δ^15^N				
Intercept	9.96	0.61	16.29	< 0.001
Location	−0.90	0.36	−2.55	< 0.05
Sex	−0.42	0.24	−1.71	> 0.05
Age	0.17	0.25	0.67	> 0.05
Season				
Summer	0.38	0.37	1.03	> 0.05
Autumn	−0.26	0.39	−0.65	> 0.05
Winter	−0.46	0.45	−1.03	> 0.05

**TABLE 3:** The influence of the random effect, location ID, for each δ^13^C and δ^15^N from fox vibrissae, using Linear Mixed‐Effect Models (LMMs).

	Variance	SD	Total variance	% Total variance
δ^13^C				
Location ID	0.41	0.64	0.75	55
Residual	0.34	0.59		45
δ^15^N				
Location ID	0.67	0.82	1.45	46
Residual	0.78	0.88		54

### 3.2 The Proportion of Anthropogenic Food in Rural and Urban British Fox Diet

Prior to modelling, significant differences between the δ^13^C and δ^15^N of each food group were revealed across each pairwise comparison (Table 4).

**TABLE 4:** *F*‐statistics (and degrees of freedom) of the PERMANOVA pairwise comparisons between potential food sources (comparisons where *p* < 0.05 are in bold).

	Human food	Pet food	Mammals	Birds	Invertebrates	Fruit
Human food	—	—	—	—	—	—
Pet food	**32.37 (1, 223)**	—	—	—	—	—
Mammals	**207.50 (1, 220)**	**62.70 (1, 329)**	—	—	—	—
Birds	**131.90 (1, 117)**	**22.03 (1, 226)**	**11.06 (1, 223)**	—	—	—
Invertebrates	**157.20 (1, 305)**	**49.31 (1, 414)**	**148.17 (1, 411)**	**163.69 (1, 308)**	—	—
Fruit	**420.09 (1, 56)**	**96.44 (1, 165)**	**490.57 (1, 162)**	**503.15 (1, 59)**	**123.88 (1, 247)**	—

**FIGURE 3:**
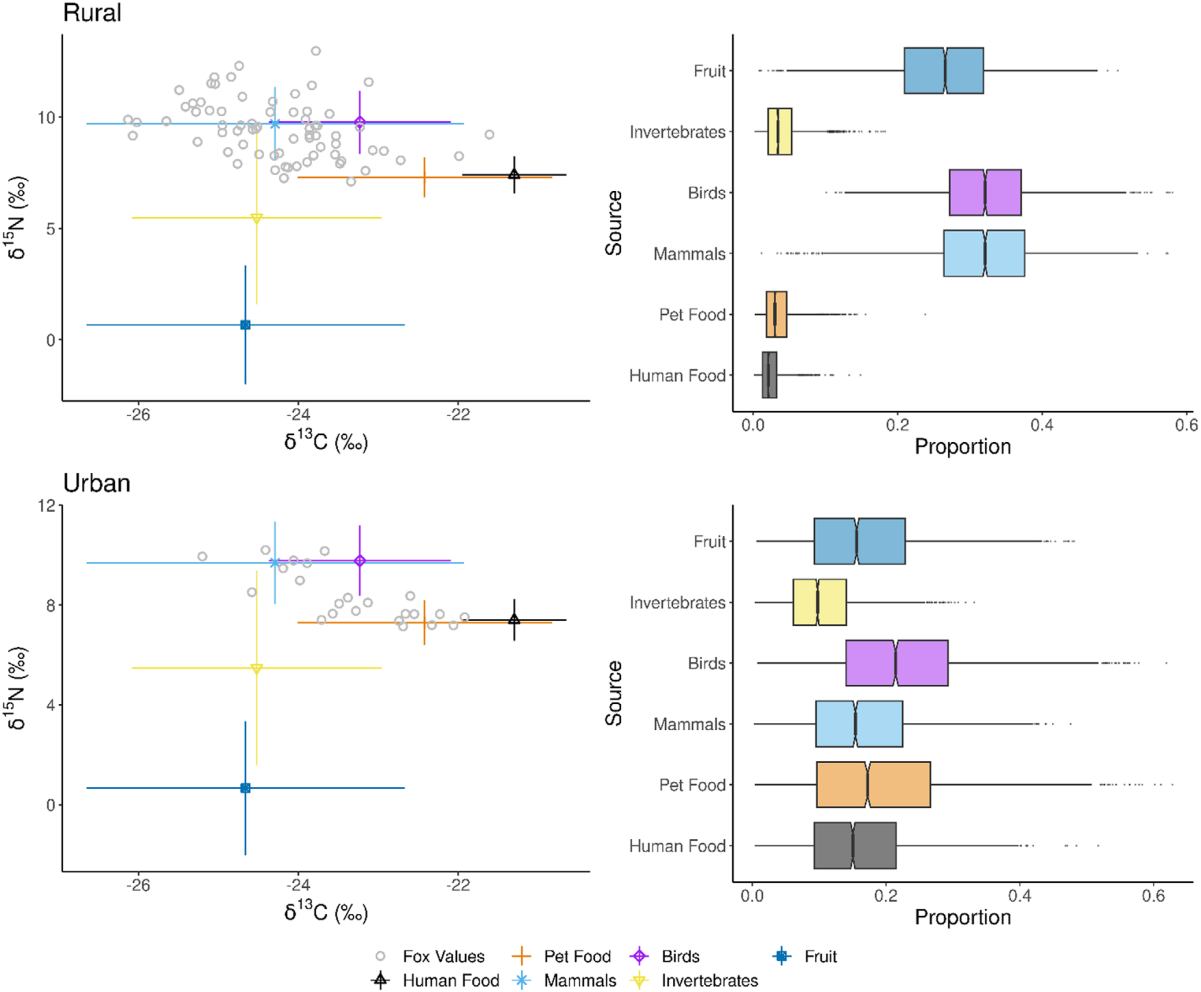
The δ^13^C and δ^15^N of the fox samples (rural and urban) and the food groups, alongside the 95% credible intervals of each food group for both rural and urban foxes, ranging from 2.5% to 97.5%, with the box centre and box edges representing 50%, 25% and 75%, respectively.

The Bayesian mixing model showed that human and pet food (anthropogenic food) each contributed similarly to the urban fox diet, constituting (as percentages) a combined 34.6% of their diet compared to 6.0% in rural fox diets. Birds were the largest dietary source for both urban and rural foxes, despite a lower portion in the urban fox diet. Mammals formed a similar proportion of the rural fox diet alongside birds but formed a lower amount of the urban diet compared to anthropogenic food (Table 5). Figure 3 shows the food sources with the spread of rural and urban fox isotope values, including the respective 95% credible intervals for each food source.

**TABLE 5:** The mean proportion (± SD) of each food group consumed by rural and urban foxes.

Food item	Rural (SD)	Urban (SD)
Human food	0.025 (0.016)	0.157 (0.083)
Pet food	0.035 (0.023)	0.189 (0.114)
Mammals	0.317 (0.082)	0.163 (0.087)
Birds	0.322 (0.074)	0.221 (0.108)
Invertebrates	0.040 (0.026)	0.104 (0.055)
Fruit	0.262 (0.081)	0.165 (0.091)

**FIGURE 4:**
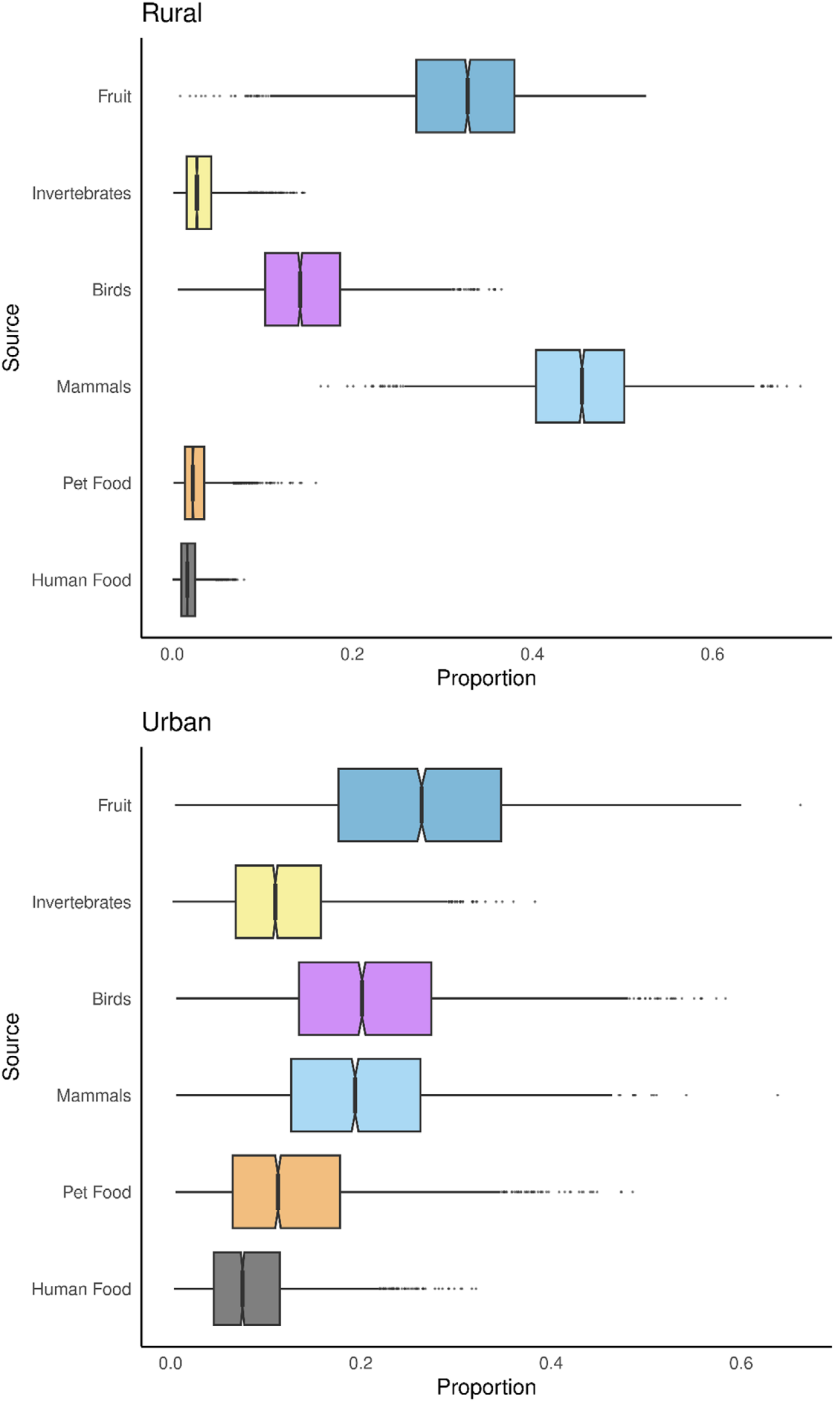
The 95% credible intervals of the food group proportions using the alternative TDFs from Roth and Hobson (2000) for both rural and urban foxes. The intervals range from 2.5% to 97.5% with the box centre and box edges representing 50%, 25% and 75%, respectively.

In contrast, the mixing model which used the TDFs from Roth and Hobson (2000) demonstrated a comparable amount of anthropogenic food in rural fox diet (4.6%); however there was a smaller proportion for urban foxes (21.4%). In this model, mammals were the largest food source for rural foxes, followed by fruit. Mammals and birds still contributed similarly to the urban fox diet, despite forming a lower proportion overall; however, fruit was the food group with the greatest proportion (Table 6). Figure 4 demonstrates the 95% confidence intervals for this model.

**TABLE 6:** The mean proportion (± SD) of each food group consumed by rural and urban foxes for the mixing model using the alternative TDFs from Roth and Hobson (2000).

Food item	Rural (SD)	Urban (SD)
Human food	0.019 (0.012)	0.085 (0.051)
Pet food	0.027 (0.018)	0.129 (0.081)
Mammals	0.453 (0.074)	0.198 (0.094)
Birds	0.146 (0.061)	0.210 (0.101)
Invertebrates	0.033 (0.022)	0.118 (0.063)
Fruit	0.322 (0.082)	0.262 (0.116)

### 3.3 Sex, Age and Season

**FIGURE 5:**
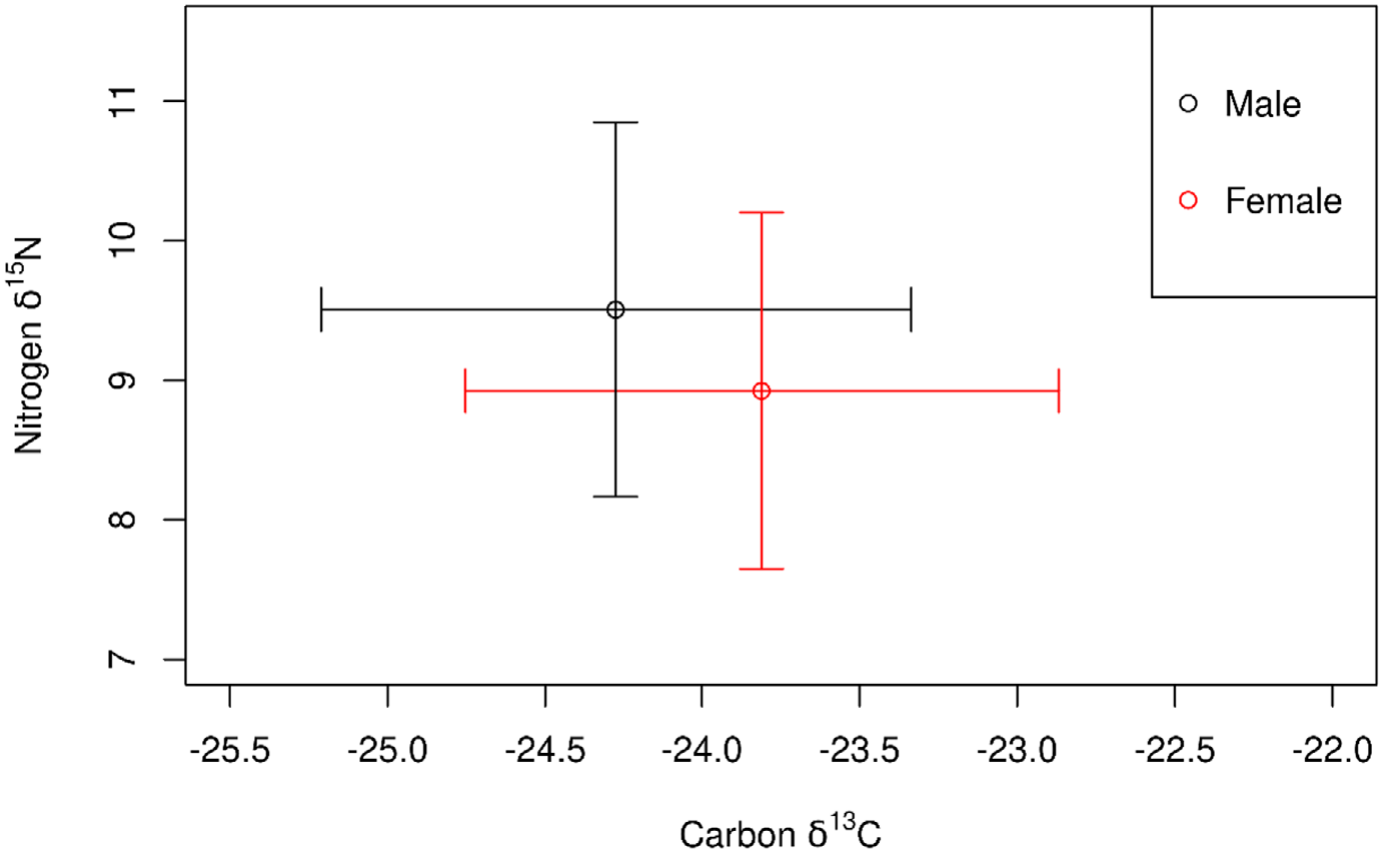
Mean (± SD) δ^13^C and δ^15^N of male (*n* = 43) and female (*n* = 50) fox vibrissae from urban and rural locations combined.

For δ^13^C, females demonstrated significantly higher values than males, however, for δ^15^N, there was no significant effect of sex (Table 2; Figure 5).

For each element, age (subadult and adult) was not significant for δ^13^C or δ^15^N (Table 2). Additionally, season was also not significant for δ^13^C or δ^15^N (Table 2).

## 4 Discussion

This study demonstrated that urban foxes had higher δ^13^C and lower δ^15^N compared to rural individuals, which is indicative of increased anthropogenic food consumption (e.g., Newsome et al. 2010). The amount of anthropogenic food (human and pet food) in the urban fox diet was estimated to be 34.6% compared to 6.0% in rural fox diets. Overall, females had higher δ^13^C than males, suggesting higher anthropogenic food use by females. Age and season had no effect on isotopic ratios.

### 4.1 Anthropogenic Food Use by Foxes in Britain

Our mixing model indicated that anthropogenic food (human and pet food) formed a larger dietary proportion in British urban foxes than rural foxes. The higher δ^13^C and lower δ^15^N of urban compared to rural foxes is a pattern demonstrated in previous studies that constrast between conspecifics in urban environments to those in areas classified as rural/less impacted by human activity. Examples include red foxes in Germany (Scholz et al. 2020), San Joaquin kit foxes ( *Vulpes macrotis mutica* ) in California, USA (Newsome et al. 2010) and caracals ( *Caracal caracal* ) in South Africa (Leighton et al. 2023). Values of δ^13^C in British anthropogenic food are lower compared to US anthropogenic food, due to the greater use of corn in the US food industry (Jahren and Kraft 2008; Hülsemann et al. 2015). Whilst this may impact the ability to distinguish between natural and anthropogenic food use, our results reinforce that anthropogenic use by foxes in Britain can be determined from SIA.

Furthermore, an additional mixing model that used the TDFs from the highly cited Roth and Hobson (2000) study led to a lower proportion of anthropogenic food in urban fox diets (21.4%). These TDFs differed slightly from the ones in our main mixing model for Δ^13^C (+0.6%); additionally, error terms were not clearly provided and therefore not included. Nevertheless, their use led to a reasonable difference in anthropogenic food dietary proportions. The TDFs in the main model more accurately reflect the fox diet, since they consider the impact of a mixed (C3 and C4) diet, which would be expected for urban foxes.

### 4.2 The Influence of Sex, Age and Season on Isotopic Values

We predicted there would be no isotopic differences between males and females; however, females demonstrated higher δ^13^C than males, which suggested their diet contained more anthropogenic food than males. We hypothesise that adult females may utilise this resource more when raising offspring. During the cubbing season in spring, reproducing females are known to provide cubs with more energetically optimised food, such as larger food items, compared to their own diet (Lanszki et al. 2023). The abundant, predictable and likely energy‐dense anthropogenic food items may constitute optimal energy intake. Indeed, females have demonstrated greater use of intentional provisioning during this period (Dorning and Harris 2017; Fawcett, Fawcett, and Soulsbury 2017). In comparison, males may not utilise anthropogenic food sources as much as females, since resident males may reduce foraging from intentional provisioning patches during the winter (Dorning and Harris 2017), as they likely spend time making extra territorial excursions in search of mating opportunities (Iossa et al. 2008). Whilst the results did not demonstrate a significant influence of sex on δ^15^N, females generally had lower values than males which may be more notable in a larger dataset.

Age did not impact fox stable isotope values, since we found no difference in the δ^13^C and δ^15^N between subadults and adults, which confirmed our prediction. Age may covary with social status, as we assume that older individuals are more likely to be dominant (Baker et al. 1998). Since dominants and subordinates use food from provisioning households differently (Dorning and Harris 2017), dissimilarities in isotopic patterns for age might be expected. However, this assumes a degree of rigidity in dominance retention that may vary by sex and population density (Iossa et al. 2009). We hypothesise that age may also play a role in the consumption of anthropogenic food more strongly in females than males. Since urban female use of anthropogenic feeding sites is greater in spring (Fawcett, Fawcett, and Soulsbury 2017; Dorning and Harris 2017) and assuming these females are raising offspring, we might expect differences in anthropogenic food use between older breeding and younger non‐breeding females. Additionally, many yearling females fail to reproduce (Iossa et al. 2008). Furthermore, studies have shown that subadults of both sexes (< 12 months old) differed in both dietary item consumption and dietary breadth compared to adult females, although there was no difference between subadults and adult males (Kidawa and Kowalczyk 2011; Forbes‐Harper et al. 2017). Nevertheless, these potential age and social status effects may be obscured isotopically by the abundance of multiple sources of anthropogenic foods in an urban environment, whereby most urban fox individuals consume anthropogenic food indiscriminately. However, there appears to at least be a difference in anthropogenic food use at the level of sex.

Our prediction that winter would have significantly lower δ^15^N and higher δ^13^C compared to the other seasons was not confirmed. Across seasons, δ^15^N was the lowest and δ^13^C was the highest in the winter, however this was not statistically significant. Despite our results, urban foxes tend to increase their anthropogenic food use during the winter to supplement their diet when natural food abundance is low (e.g., Harris 1981). Indeed, the number of visiting urban foxes to households that intentionally provide food is greatest during the winter (Dorning and Harris 2017). In more natural habitats, foxes can consume more carrion in the winter when other prey species are scarce (Goszczyńsk 1986; Sidorovich, Sidorovich, and Izotova 2006). Scholz et al. (2020) did not find the time of year to influence isotopic values in red foxes, although this may also result from analytical differences. We used a categorical predictor, season, whereas they used a continuous predictor of Julian day within a linear model.

### 4.3 Implications of Anthropogenic Food Use

In our study, foxes in urban areas and females (more than males) consumed the most anthropogenic food and are therefore more likely to be impacted by any positive or negative consequences. For example, synanthropes may experience improved body condition (i.e., energy reserves calculated using measures of body mass and skeletal morphology; Lyons et al. 2017); however, such mass increases may also be negatively associated with measures of obesity or insulin resistance (Banks et al. 2003; Leith et al. 2020). In contrast, anthropogenic food consumption in coyotes ( *Canis latrans* ) has been linked to poorer body condition, lower protein intake, mange infestation, human–conflict behaviour and greater microbiome diversity (Murray et al. 2015b; Sugden et al. 2020). The body mass and skeletal size of foxes have been shown to increase across a rural‐to‐urban gradient, although their body condition remained unchanged (Stepkovitch et al. 2019). However, research has not yet explored urban fox body condition in relation to diet using SIA. We might find, as in previous research, a subset of the population to be adversely impacted by anthropogenic food use (e.g., Banks et al. 2003; Leith et al. 2020). Alternatively, even if body condition itself is unaffected by anthropogenic food use, synanthropes may experience other health impacts such as malnutrition and oxidative stress (Bernat‐Ponce et al. 2023). Lastly, any potential impact of anthropogenic food consumption by foxes may be limited to the winter season. Whilst our results did not indicate that anthropogenic food use is potentially greater during the winter, previous research has shown otherwise (Harris 1981; Doncaster, Dickman, and Macdonald 1990; Saunders et al. 1993). Whilst such consumption may be prevalent in other seasons, a potential urban population‐level impact of anthropogenic food may be most pronounced during the winter.

### 4.4 Fruit Consumption in Red Fox Diet

Notably, fruit formed 26.2% and 16.5% of rural and urban fox diets, respectively, despite the distance between fruit and the fox samples within isotopic space. Mixing models assume equal concentrations of carbon and nitrogen from each source, which can impact estimates if violated (Phillips and Koch 2002). The inclusion of elemental concentrations accounted for the relatively lower nitrogen of fruit compared to other sources; the exclusion of which resulted in negligible dietary fruit proportions. In previous urban fox diet studies in Britain, fruit formed between 5% and 9% of the diet (Harris 1981; Doncaster, Dickman, and Macdonald 1990; Saunders et al. 1993). Fruit (under the category of ‘plant’) formed approximately 37% (frequency of occurrence) in fox diets across Europe (Soe et al. 2017). On a continental scale, urbanisation (i.e., human footprint index) was positively associated with fruit consumption (Castañeda et al. 2022). Whilst our findings demonstrated a lower proportion of fruit in urban areas compared to rural fox diets, our mixing model results fall within a reasonable estimate. Additionally, previous British urban fox diet studies may have under‐reported fruit proportions as a consequence of sampling method biases (Castañeda et al. 2022). Notably, fruit proportions increased when using the TDFs from Roth and Hobson (2000), which again demonstrates the importance of TDFs in modelling.

### 4.5 Limitations and Future Research

There are multiple methods for defining urbanisation; we followed the same impervious surface density method as Scholz et al. (2020) to produce comparative results. Future research could consider the relationship between additional urban metrics and fox isotopes. Metrics might include developed open spaces (such as parks and gardens), since δ^13^C can be positively correlated with open space (Handler, Lonsdorf, and Ardia 2020), as range size can be influenced by residential garden size and connectivity (Baker et al. 2000; Tolhurst et al. 2020). Furthermore, more data are required to sufficiently examine interactions between location and sex. Practically, more rural foxes were collected than urban foxes due to the biased nature of fox control, the majority of which was conducted by rural gamekeepers. The low urban to high rural fox ratio in Scholz et al. (2020) likely reflects the difficulty in attaining urban fox samples.

The potential interaction between sex and age in anthropogenic food use could be further elucidated by treating age as a continuous variable rather than a binary variable (subadult and adult), since dominants have been shown to live around 4.5 and subordinates 2.1 years on average (Baker et al. 1998). Future work that treats age in years may notice potential age effects in anthropogenic food consumption in foxes at least older than 2 years, due to an increased likelihood of dominant status.

Some of our isotopic data points were plotted outside the convex hull defined by the food sources, where rural data points had higher δ^15^N. This could suggest the existence of an unconsidered food group utilised by the foxes or that the food group isotopic range was not fully representative. Alternatively, these data points may result from factors such as fertilisers used in arable farming, which inflate nitrogen values (Schmidt and Ostle 1999); these are location‐dependent and difficult to consider at the national scale. Therefore, future work could also explore the impact of location on isotopic variation in wildlife food sources across Britain.

Lastly, it should be clearly stated that anthropogenic food use in foxes is isotopically defined by the presence of C_4_ plants (i.e., corn/maize) in these resources (Jahren and Kraft 2008; Newsome et al. 2010), which does not cover the full range of potential anthropogenic foods (e.g., garden or allotment produce; Bateman and Fleming 2012). Whilst C_4_‐heavy anthropogenic food is most likely associated with poor nutrition and negative health outcomes (Cordain et al. 2005; Banks et al. 2003; Leith et al. 2020), SIA does not cover the full range of possible anthropogenic resources in fox diets.

## 5 Conclusion

SIA is useful for determining the degree of anthropogenic food use between urban and rural foxes in Britain. It is an alternative approach to visual diet tracing methods, reflecting diet over longer time periods, and can more easily identify highly digestible food sources (i.e., anthropogenic food). Our study indicates that urban foxes in Britain incorporated more anthropogenic food in their diet than their rural conspecifics. Additionally, anthropogenic food appeared to be consumed more by female foxes than males. Whilst the data appeared to suggest anthropogenic food consumption was more prevalent in winter, a larger dataset is required to confirm this isotopic pattern. If future research uncovers poor health impacts as a result of anthropogenic food consumption, the impact would likely be most pronounced in female foxes and in those that are urban‐dwelling.

## Author Contributions

**Jonathan W. J. Fletcher:** conceptualization (equal), data curation (lead), formal analysis (equal), investigation (lead), methodology (lead), project administration (equal), visualization (lead), writing – original draft (lead), writing – review and editing (equal). **Simon Tollington:** formal analysis (equal), supervision (equal), writing – review and editing (equal). **Ruth Cox:** project administration (equal), supervision (equal), writing – review and editing (equal). **Bryony A. Tolhurst:** supervision (equal), writing – review and editing (equal). **Jason Newton:** formal analysis (equal), investigation (equal), resources (equal), writing – review and editing (supporting). **Rona A. R. McGill:** formal analysis (equal), investigation (equal), resources (equal), writing – review and editing (supporting). **Paul Cropper:** investigation (equal), project administration (supporting), resources (equal), writing – review and editing (supporting). **Naomi Berry:** investigation (equal), resources (equal), writing – review and editing (supporting). **Krishnaveni Illa:** investigation (equal), project administration (supporting), resources (equal), writing – review and editing (supporting). **Dawn M. Scott:** conceptualization (equal), methodology (equal), project administration (supporting), supervision (lead), writing – original draft (equal), writing – review and editing (equal).

## Disclosure

Statement on Inclusion: The research on this British species was conducted by a team from across Britain, representing appropriate experiential backgrounds and skills.

## Conflicts of Interest

The authors declare no conflicts of interest.

## Data Availability

Data will be made available to reviewers and editors. Public access will be granted upon publication (DOI: https://figshare.com/s/56f3eb0fd8e62e489b0e).

## Appendix A

**TABLE A1:** The number of data points for each variable overall and within the main variable of location (rural/urban).

Variable	Overall (*n* = 93)	Rural (*n* = 70)	Urban (*n* = 23)
Sex	Male: 43	Male: 33	Male: 10
	Female: 50	Female: 37	Female: 13
Age	Young Adult: 45	Young Adult: 36	Young Adult: 9
	Aged Adult: 47	Aged Adult: 34	Aged Adult: 13
	Unknown: 1	Unknown: 0	Unknown: 1
Season	Spring: 21	Spring: 16	Spring: 5
	Summer: 29	Summer: 27	Summer: 2
	Autumn: 23	Autumn: 15	Autumn: 8
	Winter: 18	Winter: 10	Winter: 8
	Unknown: 2	Unknown: 2	Unknown: 0
